# Seasonality of Aerosol Sources Calls for Distinct Air Quality Mitigation Strategies

**DOI:** 10.3390/toxics10030121

**Published:** 2022-03-03

**Authors:** Chunshui Lin, Darius Ceburnis, Colin O’Dowd, Jurgita Ovadnevaite

**Affiliations:** 1 Centre for Climate and Air Pollution Studies, Ryan Institute, School of Physics, National University of Ireland Galway, University Road, H91 CF50 Galway, Ireland; chunshui.lin@nuigalway.ie (C.L.); ceburnis.darius@nugalway.ie (D.C.); 2 State Key Laboratory of Loess and Quaternary Geology and Key Laboratory of Aerosol Chemistry and Physics, Institute of Earth Environment, Chinese Academy of Sciences, Xi’an 710061, China

**Keywords:** rolling PMF, source apportionment, air pollution, submicron aerosol, aerosol chemical speciation monitor

## Abstract

An Aerosol Chemical Speciation Monitor (ACSM) was deployed to investigate the temporal variability of non-refractory particulate matter (NR-PM_1_) in the coastal city of Galway, Ireland, from February to July 2016. Source apportionment of the organic aerosol (OA) was performed using the newly developed rolling PMF strategy and was compared with the conventional seasonal PMF. Primary OA (POA) factors apportioned by rolling and seasonal PMF were similar. POA factors of hydrocarbon-like OA (HOA), peat, wood, and coal were associated with domestic heating, and with an increased contribution to the OA mass in winter. Even in summer, sporadic heating events occurred with similar diurnal patterns to that in winter. Two oxygenated OA (OOA) factors were resolved, including more-oxygenated OOA and less-oxygenated OOA (i.e., MO-OOA and LO-OOA, accordingly) which were found to be the dominant OA factors during summer. On average, MO-OOA accounted for 62% of OA and was associated with long-range transport in summer. In summer, compared to rolling PMF, the conventional seasonal PMF over-estimated LO-OOA by nearly 100% while it underestimated MO-OOA by 30%. The results from this study show residential heating and long-range transport alternately dominate the submicron aerosol concentrations in this coastal city, requiring different mitigation strategies in different seasons.

## 1. Introduction

Atmospheric aerosol or particulate matter (PM) plays an important role in the regional and global climate system [1]. It also adversely affects local and regional air quality [2], causing visibility reduction [3] and impairing human health [4]. Globally, atmospheric particulate pollution leads to millions of premature deaths every year [5,6]. Several studies [7,8,9] showed that current levels of PM2.5 observed in European cities are exceeding the guidelines recommended by the World Health Organization [10]. Developing cost-effective mitigation strategies requires a better understanding of the chemical composition and sources of PM, as well as the corresponding temporal variability (e.g., seasonal, diurnal, etc.) [11,12,13].

Organic aerosol (OA) is one of the most important components of PM, constituting 20–90% of the submicron PM (PM_1_) mass [14]. Atmospheric OA contains thousands of organic molecules, which have been reported to be more toxic than inorganic aerosol, e.g., sulfate and nitrate [9,15]. OA can be directly emitted from emission sources of e.g., traffic [16] and biomass burning [17] and was, thus, termed as primary OA (POA), while secondary OA (SOA) is produced in the atmosphere from the oxidation of the precursor gases of volatile organic compounds [18,19,20,21]. Recently, the development of the Aerodyne Aerosol Chemical Speciation Monitor (ACSM) [22] has enabled a near real-time sampling of bulk OA mass spectrum on a routine basis at urban air quality (AQ) monitoring stations (e.g., the AQ network at www.macehead.org accessed on 1 February 2022), in addition to other inorganic non-refractory PM_1_ species (NR-PM_1_) (i.e., nitrate, sulfate, ammonium, and chloride). Combined with positive matrix factorization (PMF) [23], OA factors of e.g., hydrocarbon-like OA (HOA), biomass burning OA (BBOA), and oxygenated OA (OOA) are identified and linked to their corresponding emission sources and/or atmospheric processes [14,24,25]. However, PMF assumes that OA factor profiles are static, which is contradictory to the fact that OA are constantly evolving in the atmosphere. Therefore, source apportionment of OA is complicated as an extended period (e.g., months to years) of the OA mass spectrum is collected. Usually, a season-wise OA source apportionment is performed to at least account for the seasonality of OA factor profiles [26,27], although substantial uncertainties can also be caused by this conventional PMF approach because any short-term (days to weeks) variations will still be averaged out even in seasonal PMF.

Galway city lies on the west coast of Ireland and is the fourth most populous city in the Republic of Ireland, with a population of ~80,000 according to the 2016 census (www.cso.ie; accessed on 1 December 2021). Through the short-term campaign using an ACSM in combination with PMF, we reveal that primary emissions from residential heating can cause severe pollution in winter [17], while secondary aerosol from long-range transport is the major source during summer [28]. However, long-term sampling of submicron aerosol in this city is currently lacking, limiting our understanding of the seasonal variability of the chemical composition and OA factors. 

In this study, an extended ACSM measurement was conducted in Galway city from February to July 2016, covering the season of winter, spring, and summer. To account for the temporal variation in the OA source profiles, a novel PMF strategy (see Section 2.3; [29]) with a short rolling time window was applied (henceforth referred to as rolling PMF). The result from the rolling PMF is compared with conventional seasonal PMF analysis. Furthermore, the potential geographic origins of POA and SOA factors are investigated as a function of wind speed and wind direction using polar plots. 

## 2. Materials and Methods

### 2.1. Sampling Site

Ambient aerosol was sampled at a residential site in Galway city (Figure S1) from 5 February 2016 to 31 July 2016, covering the season of winter (February), spring (March, April, and May), and summer (June and July) and representing the first extended (6 months) measurement of the chemical composition of submicron aerosol in this city. The sampling site is 1 km to the northwest of the city center and is 200 m away from the nearest traffic road (Figure S1). Measurements were conducted in the Center of Climate and Air Pollution Studies (6 m above the ground) on the campus of the National University of Ireland Galway (NUIG, 53.28° N; 9.06° W). The sampling site is considered as an urban background site as it is located in a residential area, with no immediate strong traffic impacts. 

### 2.2. Instrument

A quadrupole ACSM (Aerodyne Research Inc., Billerica, MA, USA) was deployed to measure the major components of non-refractory submicron aerosol (NR-PM_1_) including organics, sulfate, nitrate, ammonium, and chloride with a time resolution of 30 min [22]. The design and working principles of ACSM have been detailed in Ng, et al. [22]. Briefly, ambient air was pumped in at a flow rate of 3 L min^−1^ and was subsampled into the ACSM system through a critical orifice of 100 μm in diameter at a flow rate of 85 cc min^−1^. The sampled aerosol particles were focused into a narrow beam by an aerodynamic lens system and were directed to impact upon a hot tungsten surface of approximately 600 °C, where the non-refractory particles were flash vaporized and were subsequently ionized using an electron impact at 70 eV. The resulting ions were detected by a quadrupole mass spectrometer. The ensemble mass spectrum was determined by the difference between the total ambient signal and the particle free air which was controlled using an automated three-way switching valve that is switched between the sample and particle-free air measurement every ~30 s. Using the fragmentation table [30], the measured ions were attributed to the five major NR-PM_1_ species: OA, sulfate, nitrate, ammonium and chloride. ACSM was calibrated using size-selected (300 nm) particles of the atomized ammonium nitrate and ammonium sulfate particles. An ionization efficiency (IE) of 2.9 × 10^−11^, a relative ionization efficiency (RIE) of 0.6 for sulfate, and an RIE of 4.6 for ammonium were determined through calibration. For ambient measurement, a collection efficiency of 1 was applied, consistent with our previous studies at the sampling site [17,28] and representing the low limit of the NR-PM_1_ concentrations. Note that the unit mass resolution of ACSM makes it hard to separate different ions at the same nominal *m*/*z* (e.g., C_2_H_3_O and C_3_H_7_ at *m*/*z* 43). When combined with PMF, OA factors with similar mass spectral patterns are prone to mixing without extensive analysis and evaluation.

### 2.3. Complementary Data 

An Aethalometer (AE-33; Magee Scientific, Berkeley, CA, USA) was deployed alongside ACSM to measure the concentration of the equivalent black carbon (BC) only from 5 February to 9 April 2016 with a time resolution of 1 min [31]. Meteorology data including temperature, wind speed, wind direction, and relative humidity (RH) with 1 h time resolution were obtained from the nearest Met station (20 km to the east), managed by the Irish National Meteorological Service (Met Éireann at met.ie accessed on 1 February 2022). All time reported in this study is local time.

### 2.4. Source Apportionment of Organic Aerosol

Positive matrix factorization (PMF) with the multilinear engine (ME-2) [23,32] was employed to analyze the contributions of different sources and/or atmospheric processes to the measured OA on the interface of SoFi (version 6.G), an Igor Pro package developed by Canonaco, et al. [24]. PMF can be defined as

X = GF + E,

where X is the time series of the measured organic ions in matrix notation, which can be approximated by the product of G (factor time series) and F (factor mass spectral profile), while E is the model residual with the same dimension as X. Although the PMF model requires all the elements of the model outputs to be nonnegative, it suffers from rotational ambiguity where different combinations of G and F (i.e., different PMF solutions) have similar levels of residuals but not all of the PMF solutions are physically meaningful. Constraining PMF using the ME-2 algorithm can guide the model towards an environmentally reasonable solution based on priori information e.g., known factor profiles or time series. The *a* value approach, a constraining strategy enabled by SoFi [24], allows for different degrees (ranging from 0 to 1) of variation from the anchoring profiles, enabling the comprehensive exploration of the solution space. Conventional PMF or ME-2 analysis is conducted over the entire campaign period or at least season-wide, assuming that the OA factor profiles are static, which can cause large uncertainties given that OA is constantly evolving in the atmosphere. Performing PMF on a smaller time window (e.g., days) to roll over the whole dataset can capture the temporal variation of the source profiles [18]. The rolling PMF mechanism with the bootstrap strategy, enabled by SoFi Pro [29], was applied to the current dataset to evaluate the time-dependent source apportionment of organic aerosol and the corresponding uncertainties.

In this study, the standard ACSM software of acsm_local_1611 software (Aerodyne Research Inc., Billerica, MA, USA) was used to prepare the PMF input matrix. The input matrix contained 7, 401 time points and 69 organic ions with *m*/*z* in the range of 16–100. Of these ions, CO_2_^+^ -related ions (*m*/*z* 16, 17, and 18) were excluded prior to a PMF analysis but were reinserted into the OA factor mass spectra after the PMF analysis using the fixed ratios based on the fragmentation table [30]. Note that organic ions of *m*/*z* of 12, 13, and 15 and *m*/*z* of >100 were not considered during the PMF analysis due to the low signal-to-noise ratio, while *m*/*z* 29 were biased by the interference with air signals and was, thus, not considered. 

Unconstrained or free PMF was firstly performed to evaluate the OA factors on a season-wide basis. Free PMF solutions resulted in highly mixed factor profiles due to the temporal covariation of the OA factors, i.e., all peaking at a similar time corresponding to the domestic heating emission time (Figure S2), which makes it difficult for unconstrained PMF to separate them unambiguously. For example, during winter, the mass spectral profiles for the unconstrained factors had some missing *m*/*z*’s of e.g., 41, 43, or 44 (Figure S2), which was, as a result, attributed to other factors (i.e., mixing). Therefore, free PMF had large uncertainties in OA source apportionment. Previous studies [17,33,34] and the census data in Ireland [35] show the combustion of heating oil, peat, wood, and coal are the candidate factors of the heating emissions. Based on this priori information, the reference mass spectral profiles of hydrocarbon-like OA (HOA [36]) was constrained with a random *a* value in the range of 0–0.1 with a step of 0.05 (i.e., *a* value of 0, 0.05, 0.1 was randomly constrained [29]), while the reference peat, wood, and coal burning OA profiles were used as the anchoring profiles using the limits approach (an option in SoFi within the tab of a value approach) following our previous study [37]. At the same time, 100 bootstrap runs were performed to evaluate the uncertainties of the constrained PMF. Specifically, the bootstrapping resampling strategy created a new input matrix by randomly duplicating some time points while excluding others, preserving the original dimension of the input matrix. ME-2 was performed on these new input matrices. A total of 100 bootstrap runs were averaged as the optimized solution, with the variation reflecting the model uncertainty. During the summer, the fraction of *m*/*z* 60 (*f60*) in the total organics exceeded the reference background level (0.3%; Figure S3) [38] and, therefore, the heating-related factors (peat, wood, and coal) were also constrained in the summer. Two oxygenated OA (OOA) factors, i.e., one more oxidized OOA (MO-OOA) and one less oxidized OOA (LO-OOA) were left unconstrained. 

The rolling PMF mechanism [29] with a time window of 14 days and a step of 1 day was applied to the entire dataset to account for the temporal variabilities of the factor profiles over time. In other words, instead of running PMF for different seasons separately, the smaller time window (i.e., 14 days) for the rolling PMF moved with daily steps over the entire dataset, allowing the PMF model to adapt the factor profiles across different observation periods gradually. The rolling window size of 14 days was suggested by Canonaco, et al. [29] and was directly adopted in this study. As also shown in Chen, et al. [27], 14-day window size is sufficient to capture the variability of factor profiles. The same constraining strategy and bootstrap resampling techniques as the seasonal bootstrap PMF were applied to allow for a quantitative estimate of the statistical and rotational uncertainties of the rolling PMF solutions. Both the seasonal bootstrap PMF runs and the rolling PMF runs were selected using a list of criteria (Table S1), including the correlation with the external BC measurement (*p* value ≤ 0.05), the explained variation of *m*/*z* 60 by biomass (wood + peat) burning (*p* value ≤ 0.05), while *f44* (fraction of *m*/*z* 44 in the total organics) was used for sorting unconstrained MO-OOA and LO-OOA. All the runs that met the criteria were selected and averaged as the most optimized PMF solution, with one standard deviation representing the uncertainties of PMF modelling. 

## 3. Results

### 3.1. Chemical Composition and Seasonal Variations

**Figure 1:**
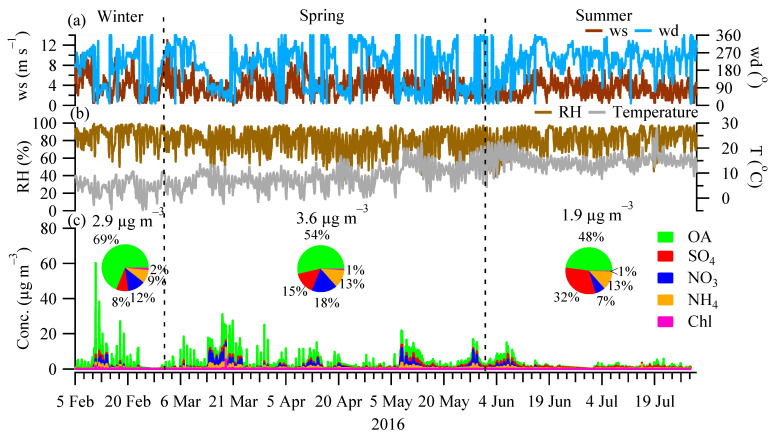
Time series of (**a**) wind speed (ws) and wind direction (wd); (**b**) relative humidity (RH) and Temperature (T); and (**c**) the major NR-PM_1_ species, i.e., organics (OA), sulfate (SO_4_), nitrate (NO_3_), ammonium (NH_4_), and chloride (Chl) from 5 February 2016 to 31 July 2016. The sampling period covered the season of winter (February), spring (March, April, and May), and summer (June and July). Inset pie charts show the relative contribution of different NRPM_1_ species, with values above showing the average concentration over different seasons.

Figure 1 shows the time series of the OA, sulfate, nitrate, ammonium, and chloride, as well as meteorological parameters (temperature, wind speed, wind direction, and relative humidity) from 5 February to 31 July 2016. Over the entire sampling period, the concentration of NR-PM_1_ (sum of OA, sulfate, nitrate, ammonium, and chloride) varied from <1.0 μg m^−3^ to 60.2 μg m^−3^, with an average of 3.0 μg m^−3^. Most NR-PM_1_ spikes (>25 μg m^−3^) occurred in the winter and spring when temperatures were relatively low (<5 °C) in relatively calm weather (wind speed < 4 m s^−1^). Figure 1 also shows different seasons had different NR-PM_1_ concentrations with drastically different contributions from the measured species. Specifically, the average NR-PM_1_ concentrations in winter (2.9 μg m^−3^) and spring (3.6 μg m^−3^) were 1.5–1.9 times higher than in summer (1.9 μg m^−3^). In winter, OA was the most dominant component of the NR-PM_1_, on average, accounting for 69% (2.0 μg m^−3^) of NR-PM_1_, followed by nitrate (12% or 0.4 μg m^−3^), ammonium (9% or 0.3 μg m^−3^), sulfate (8% or 0.2 μg m^−3^), and chloride (2% or 0.04 μg m^−3^). In spring, OA accounted for over half (54% or 1.9 μg m^−3^) of the NR-PM_1_, while nitrate and sulfate accounted for 18% (0.6 μg m^−3^) and 15% (0.5 μg m^−3^) of NR-PM_1_, respectively. In summer, sulfate showed the largest increase, on average, accounting for 32% (0.6 μg m^−3^) of NR-PM_1_ while nitrate showed the largest decrease (7% or 0.1 μg m^−3^) compared to winter and spring. At the same time, OA, on average, accounted for roughly half (48% or 0.9 μg m^−3^) of the NR-PM_1_ during summer. The changes in the chemical composition and aerosol loadings are due to the changes in aerosol sources (e.g., local vs. regional transport; discussed in Section 3.3 and Section 3.4) and the meteorological conditions of e.g., temperature. In particular, the low nitrate levels in summer were likely associated with its semi-volatile nature which remained mostly in the gas phase with a temperature of up to 28 °C in summer. Note that, during the sampling period, only one month (i.e., February) data was included in winter and, therefore, the absolute concentrations in winter can be biased, while we do not expect the relative contribution of the NR-PM_1_ species to vary a lot since organics was the major component of residential heating emissions, [17] a major aerosol source in winter (discussed in Section 3.3).

### 3.2. Diurnal Patterns of the Major Components of NR-PM_1_

**Figure 2:**
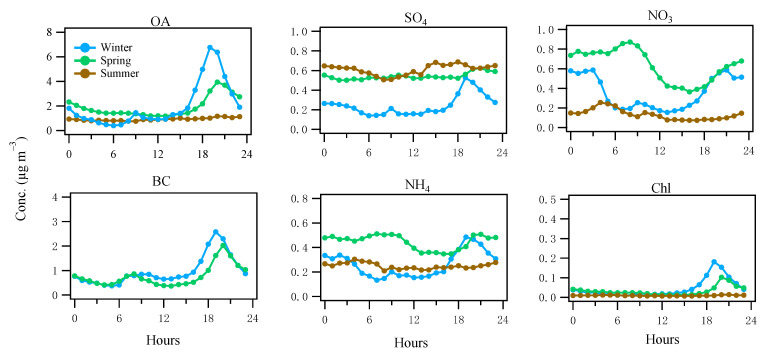
Diurnal cycles of the NR-PM_1_ species, i.e., organics (OA), sulfate (SO_4_), nitrate (NO_3_), ammonium (NH_4_), and chloride (Chl), as well as BC over winter, spring, and summer 2016. Note that BC measurements were only available in winter and spring.

Figure 2 shows the averaged diurnal patterns of NR-PM_1_ components and BC in different seasons (BC was only available in winter and spring; See Section 2.3). In both winter and spring, OA showed elevated concentrations (1.9–6.8 μg m^−3^) during the evening hours of 18:00–23:00, while, in summer, OA remained relatively flat (0.8–1.1 μg m^−3^) throughout the day. Similar to OA during winter and spring, BC, a marker of combustion emission, also showed elevated concentrations (0.9–2.6 μg m^−3^) in the evening (Figure 2). The concurrent increase in both OA and BC in the evening suggests a strong local combustion-related emission in the winter and spring, while, in summer, the flat diurnal pattern of OA indicates no strong nearby combustion sources. In addition to the evening peaks, BC also showed an increase (0.4–0.9 μg m^−3^), smaller than the evening peaks, during the morning rush hours (6:00–10:00), due to traffic emissions. In comparison, OA only showed a small bump (up to 1.4 μg m^−3^) during the morning rush hours in winter, while, in spring and summer, the morning bump was barely visible (Figure 2). Therefore, compared to the evening spikes mostly due to heating emissions (discussed in Section 3.3), such a small OA increase in the morning hours suggests traffic contribution to OA was minor, although an increasing shallow boundary layer height in the morning was also partly contributing to the decrease in OA. 

During spring and summer, sulfate was constantly elevated (0.5–0.7 μg m^−3^; Figure 2), suggesting a major source of regional transport. The slight increase in sulfate in the summer afternoon (Figure 2) suggests the photochemical formation overcame the dilution effects caused by the increasing boundary layer. As a comparison, in winter, sulfate was relatively low (0.2–0.5 μg m^−3^) and the concurrent spikes of sulfate (0.5 μg m^−3^) with BC in the evening suggests local formation associated with the solid fuel (e.g., coal [17]) combustion in winter. In summer, nitrate was relatively low (0.1–0.2 μg m^−3^) throughout the day and reached the lowest in the afternoon, corresponding to the relatively high temperature (Figure S4) in the afternoon likely causing the particle-to-gas partitioning, as well as the elevated boundary layer. In spring, nitrate increased its concentration to 0.9 μg m^−3^ in the morning, suggesting a photochemical formation after sunrise but it still showed a low valley in the afternoon, suggesting the formation was insufficient to overcome the particle-to-gas partitioning or the dilution effects caused by an increasing boundary layer height. In winter, nitrate showed a concurrent spike (0.6 μg m^−3^) with BC (Figure 2) in the evening. Nitrate remained elevated during the night which was likely formed from the precursor gases emitted from heating activities. The diurnal cycles of ammonium were closely related to those of sulfate and nitrate as a result of sulfuric and nitric acid neutralization by ammonia. Chloride showed similar spikes in the evening during spring and winter due to heating emissions, while the flat pattern of chloride in summer suggests a regional source in summer (often below the detection limit).

### 3.3. OA Source Apportionment

**Figure 3:**
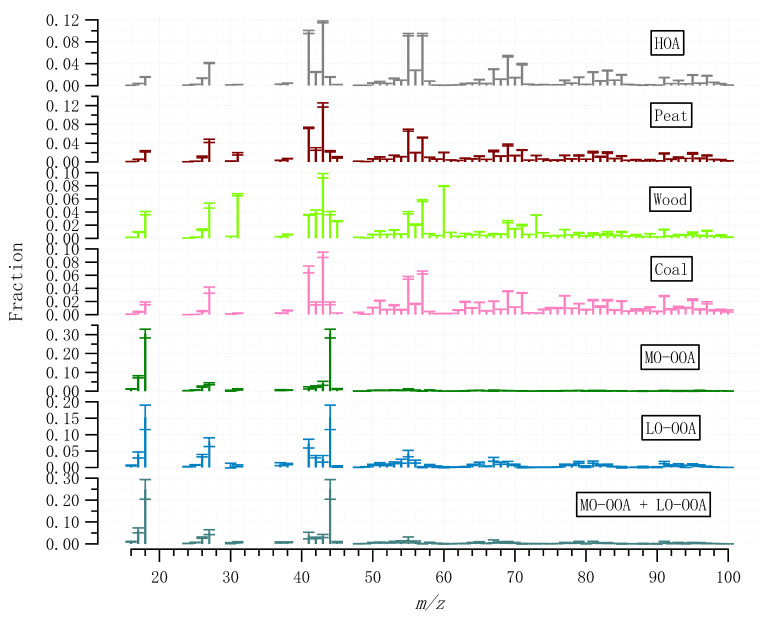
Averaged mass spectral profiles of the OA factors resolved by the rolling PMF strategy. Error bar represents one standard deviation.

**Figure 4:**
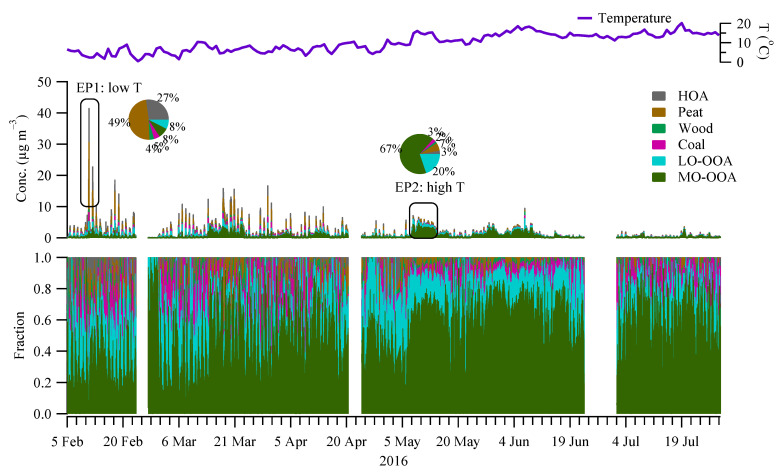
Time series of the OA factors (**mid** panel) resolved by the rolling PMF. Also shown is the time series of the temperature (**top** panel) and the relative fraction of each factor (**bottom** panel). Two episodic pollution events with one featured with low temperature (EP1) and the other with high temperature (EP2) are highlighted, with the pie charts showing the relative fraction of the factors.

**Figure 5:**
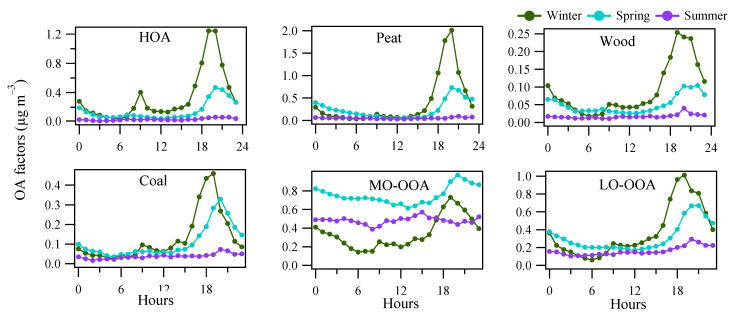
Averaged diurnal pattern of the OA factors from rolling PMF in winter, spring and summer.

A rolling PMF strategy with a 14-day time window and a step of 1-day (See Section 2.3) was applied to the entire OA matrix to evaluate the sources and/or atmospheric processes of different OA factors. In total, 53.7% of the PMF runs (9447 out of 17,600) were considered, with 0.3% (or 21) non-modelled time points. Four POA factors (HOA, peat, wood, and coal) and two OOA factors (MO-OOA and LO-OOA) were resolved. Figure 3 shows the mass spectra of the OA factors, while Figure 4 shows the corresponding time series and relative contributions. Figure 3 also shows the OOA factor, the sum of the mass-weighted MO-OOA and LO-OOA. Below, we discuss the signatures, emission strengths and formation processes for the different OA factors based on the mass spectral profiles (Figure 3), time series (Figure 4), and diurnal cycles (Figure 5).

#### 3.3.1. POA Factors

The HOA mass spectral profile is characterized by high contributions from signals at *m*/*z* of 41, 43, 55, and 57, typical of aliphatic hydrocarbons (Figure 3). The time series of HOA showed elevated concentrations in the evening concurrent with other solid fuel burning-related POA factors (Figure 4), suggesting HOA had a major source from heating emission, instead of traffic. Only <30% of the HOA in the evening can be associated with traffic assuming the evening HOA peaks had similar concertation as the morning rush hour peak (Figure 5). The small contribution of traffic to HOA can be further confirmed by comparing the difference in the diurnal pattern between weekdays and weekends (Figure S5). Specifically, during both weekdays and weekends, HOA showed a large increase from <0.1 μg m^−3^ in the afternoon to 0.4–0.5 μg m^−3^ in the evening, similar to the diurnal patterns for other solid fuel factors (Figure S5). This suggests the major HOA peaks in the evening were not affected by the change in traffic volume, which is expected to be largely reduced during the weekends. Instead, the change in traffic volume can only be reflected by changes in the HOA peaks in the morning rush hours (Figure S5) between weekdays and weekends, although the morning HOA peak was less elevated (0.1 μg m^−3^) than the evening peak (0.5 μg m^−3^). Specifically, compared to weekends, HOA showed a small peak (0.1 μg m^−3^) during the morning rush hour (~8:00) during weekdays (Figure S5). Such morning rush hour peak was missing during weekends (Figure S5), consistent with the reduced traffic during the weekends. This is also consistent with the reduced morning rush hour peak for the BC measurement (0.5 vs. 1.0 μg m^−3^), or traffic-related BC subtypes (i.e., BCtr; Figure S5) apportioned following Sandradewi, et al. [39]. As a result, the HOA to BC ratio during morning rush hours (i.e., traffic HOA/BC) was around 0.1, which is similar to the value observed at the roadside in Dublin [40] and is consistent with values found for diesel vehicular emission. The low HOA/BC ratio also suggests the primary particulate emission from traffic was mostly BC instead of HOA, resulting in a low signal for the ACSM measurement. Regarding the heating source to the HOA in the evening, among all the heating sources as surveyed by the Central Statistics Office in Ireland (CSO [35]), heating oil combustion was likely the source of HOA since oil is comprised of a mixture of hydrocarbons. In winter and spring, HOA was more elevated than in summer (Figure 5), due to more consumption of heating oil compared to summer. Moreover, the low temperatures and shallow boundary layer in winter and spring were also partly contributing to the elevated HOA concentration. 

Similar to HOA, the mass spectral profiles for solid fuel burning OA factors (peat, wood, and coal) are also characterized by large contributions from aliphatic hydrocarbons-related fragments, with elevated signals at *m*/*z* 41, 43, 55, and 57 (Figure 3). However, compared to HOA, the diurnal patterns for all solid fuel factors showed relatively low concentration during the morning rush hours (i.e., <0.1 μg m^−3^ for solid fuel factors vs. up to 0.4 μg m^−3^ for HOA; Figure 5), suggesting peat, wood, and coal burning OA were well separated from HOA. Among the solid fuel factors, the key difference in the mass spectra is the fraction of *m*/*z* 60 in the total OA signal (i.e., *f60*), which was in the order of wood > peat > coal (Figure 3). In ACSM, the signal for the organic fragment at *m*/*z* 60 (mostly C_2_H_4_O_2_^+^ [41]) was due to the fragmentation of anhydrosugars of e.g., levoglucosan which is from the combustion of cellulose/hemicellulose. Raw wood, peat, and coal had different levels of cellulose content as reflected in the different levels of *f60* in the ACSM mass spectra. 

For all solid fuel factors, they all show elevated concentrations during the evening with higher values in winter (0.2–2.0 μg m^−3^) than in spring (0.1–0.7 μg m^−3^) and summer (<0.1 μg m^−3^; Figure 5). Although solid fuel factors were low (<0.1 μg m^−3^) in summer, the diurnal patterns still showed slightly elevated concentrations in summer evenings. The use of solid fuel burning in the summer was confirmed with the trend of *f60* (Figure S3), showing higher values than the suggested background level [38]. Considering that the temperatures in some summer evenings dropped below 10 °C, sporadic combustion of solid fuel burning was expected.

**Figure 6:**
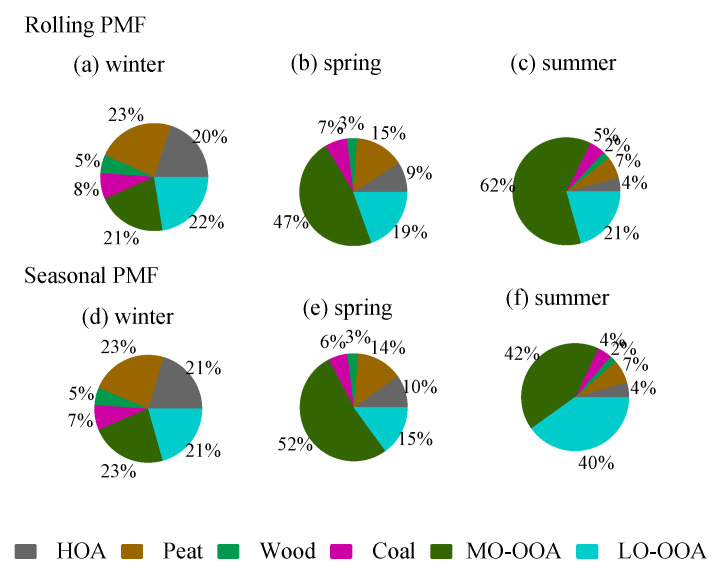
Comparison of the OA factor fractions in winter, spring, and summer apportioned by the rolling PMF (**a**–**c**) and seasonal PMF (**d**–**f**).

The rolling PMF results show that the four POA factors (HOA + Peat + Wood + Coal) together accounted for 57% of the total OA in winter (Figure 6), higher than 34% in spring, and 17% in summer. Among all the POA factors, peat burning OA was the largest factor, which accounted for 6–23% of OA in different seasons (Figure 6), followed by HOA (4–20%), Coal (5–8%), and wood (2–5%). The dominance of peat burning was more evident during the episodic pollution event (EP1) with low temperatures (<5 °C) (Figure 4), where peat accounted for nearly half (49%) of OA mass. In contrast, during EP2 with the high temperature (>15 °C), peat only accounted for 7% of the OA (Figure 3), with the majority coming from OOA factors (discussed later).

#### 3.3.2. OOA Factors

MO-OOA featured a higher fractional contribution at *m*/*z* 44 (i.e., *f44,* mostly from CO_2_^+^ [42]) than LO-OOA (Figure 3), consistent with the fact that MO-OOA was more oxidized than LO-OOA. In winter, both MO-OOA and LO-OOA showed an increased concentration in the evening (0.7–1.0 μg m^−3^) compared to 0.1–0.4 μg m^−3^ during the day (Figure 5), suggesting both OOA types had a significant contribution from heating emissions likely due to the nighttime aging processes in a shallow boundary layer [43]. In comparison, in spring and summer, MO-OOA was relatively elevated (0.4–0.9 μg m^−3^) throughout the day (Figure 5), suggesting a major contribution from regional transport, which is further supported by the wind rose analysis (see Section 3.4), while LO-OOA still shows an increase in the evening although at a smaller magnitude (0.3 μg m^−3^) in summer than in winter (1.0 μg m^−3^). Therefore, a part of LO-OOA is also associated with night-time aging of residential heating emissions in summer. 

On average, the total OOA (MO-OOA + LO-OOA) had a higher contribution (83%) to the total OA in summer (Figure 6) than in spring (66%) and winter (43%). In spring and summer, MO-OOA contributed more to the total OA (47–62% of OA) than LO-OOA (19–21% of OA), while, in winter, MO-OOA and LO-OOA had comparable contributions (21–22%) to the total OA. The large contribution from OOA was more evident during the EP2 at relatively high temperatures where the sum of OOA contributed 87% of OA, with 67% of OA attributed to MO-OOA and 20% attributed to LO-OOA (Figure 4). 

#### 3.3.3. Comparison between Rolling and Seasonal PMF

**Figure 7:**
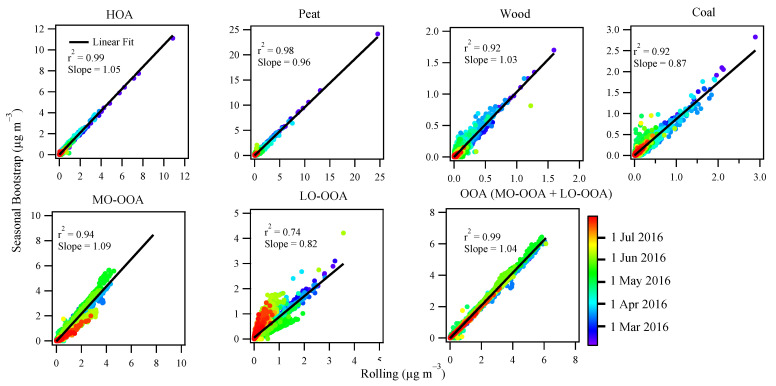
Scatter plot between the time series of the OA factors resolved by the seasonal PMF (y-axis) and rolling PMF (x-axis), color coded by date. The correlation r^2^ and slope for the linear fit are also shown for each factor.

Figure 6 compares the season-wise OA source apportionment results between the rolling PMF strategy and the seasonal PMF, while Figure 7 shows the scatter plot between the time series of the OA factors resolved from the two different PMF approaches. For POA factors, the source apportionment results between the two PMF approaches are very similar, showing roughly the same fractional contributions for different POA factors (Figure 6). Moreover, the time series for the POA factors are well correlated between the two PMF approaches with r^2^ > 0.9 and slope close to unity (Figure 7). The consistency for POA factors was expected because POA factors were constrained, although with different PMF windows (i.e., rolling 2 weeks vs. season-wise). Consistently, the mass spectra of the POA factors from the two different PMF approaches were very similar, close to the 1:1 line (Figure S7).

Compared to POA, both LO-OOA and MO-OOA factor profiles showed large differences, largely deviated from the 1:1 line as shown in Figure S7, although both PMF approaches had similar levels of the scaled residuals (Figure S8). Figure S9 shows the OOA (sum of LO-OOA and MO-OOA) factor from the rolling PMF was adapted to the measured f44/f43 (i.e., fraction of *m*/*z* 44 or 43 in the total organics), while the conventional seasonal PMF had a near static f44/f43 value, failing to capture the variation of measured OA. Moreover, for the seasonal PMF, the time series of MO-OOA was well correlated with LO-OOA (r^2^ = 0.76; Table S2), suggesting the separation between MO-OOA and LO-OOA was less confident for the seasonal PMF than the rolling PMF where the correlation between MO-OOA and LO-OOA was poorer (r^2^ = 0.54). Therefore, the OOA factors apportioned by the rolling PMF were more physically reasonable than the conventional seasonal PMF. 

Despite the differences in factor profile for the unconstrained MO-OOA and LO-OOA, both PMF approaches showed a similar fractional contribution (Figure 6) in winter and spring. However, in summer, large discrepancies were seen between the two PMF approaches (Figure 7). Specifically, MO-OOA was apportioned to account for 62% of the OA in summer by rolling PMF, while it was only 42% by seasonal PMF. LO-OOA was 21% of OA in summer by rolling PMF, while it was 40% by seasonal PMF. In other words, LO-OOA was overestimated by roughly 100%, while MO-OOA was underestimated by roughly 30% by seasonal PMF when compared to the rolling PMF. Therefore, the large discrepancies (i.e., 62% vs. 42% and 21% vs. 40%) between the two approaches suggest large uncertainties can be associated with the unconstrained OOA factors.

### 3.4. Spatial Origins of Primary and Secondary Aerosols: Local vs. Regional

**Figure 8:**
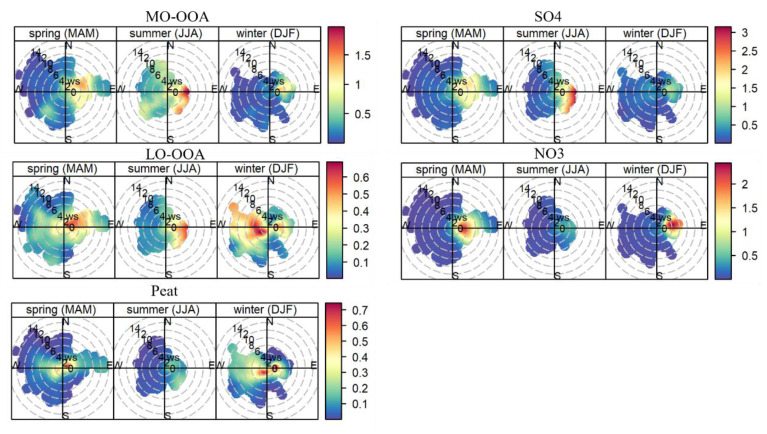
Polar plot of hourly averaged MO-OOA, LO-OOA, peat (for the rolling PMF), SO_4_, and NO_3_ as a function of wind speed (radial axis, m s^−1^) and wind direction during spring, summer, and winter, color-coded by respective concentrations (μg m^−3^). The polar plots were plotted using openair package with R software (version 4.1.2) [45].

Figure 8 shows the wind speed and wind direction-dependent concentrations for the major POA factor (peat), OOA factors (i.e., MO-OOA and LO-OOA), resolved from the rolling PMF, and secondary inorganic species (sulfate and nitrate) in spring, summer, and winter. The frequency of wind speed and wind direction (i.e., wind rose) during different seasons is given in Figure S10. During the sampling period, westerly wind (i.e., the marine air masses) with wind speed up to 13 m s^−1^ was the most frequent wind (>60%), while easterly wind, passing over inland Ireland, occurred at a lower frequency (15–40%) with relatively low wind speeds (0–6 m s^−1^). Due to the frequent clean marine air masses, the average NR-PM_1_ concentration was relatively low, as discussed in Section 3.1. However, when the weather became relatively calm with low wind speeds (<4 m s^−1^), the major POA factor of peat burning OA increased its concentrations (see the hot spot in Figure 8). The polar plot of the POA factor of Peat burning OA is similar to BC (Figure S11) with higher concentrations associated with low wind speeds. Therefore, the POA factor was mainly associated with local emissions, causing episodic pollution events as discussed previously. 

Compared to primary OA factors, the long-range transport of MO-OOA in spring and summer was evident with high concentrations (>1.5 μg m^−3^) at a wind speed of up to 6 m s^−1^ blowing from the east to south side of the sampling site (Figure 8). Moreover, even in marine air masses in summer, MO-OOA also showed slightly elevated concentration (0.5–1.5 μg m^−3^) in the westerly wind at high wind speeds (>8 m s^−1^), likely due to the transport of secondary OA formed from the oxidation of biogenic volatile organic compounds, due to active marine biological activities in summer [44]. A similar pattern is also found for sulfate, a part of which (0.5–1.5 μg m^−3^) was also associated with westerly wind although the highest sulfate (over 3 μg m^−3^) was associated with south to the easterly wind in summer (Figure 8). In winter, high MO-OOA concentrations were associated with low wind speed because MO-OOA in winter was also derived from the aging of local emissions as discussed in Section 3.3. Similar to MO-OOA, the polar plot of LO-OOA also showed features of local and regional origins. Nitrate, however, due to its semi-volatile nature, its long-range transport feature is not as straightforward as sulfate, likely because most nitrate underwent particle-to-gas partition with high temperatures in summer. Figure S12 shows the polar plots of MO-OOA and LO-OOA for the seasonal PMF solutions. The time series of the OOA factors from seasonal and rolling PMF were well correlated (r^2^ > 0.70; see Figure 7) and therefore, showed a similar pattern but with different magnitude (see the color bar differences between Figure S12 and Figure 8).

## 4. Discussion

Through the deployment of ACSM for an extended period (6 months) and in combination with the rolling PMF analysis, residential heating from solid fuel burning (wood, peat, and coal) was found to be one of the major OA sources in cold seasons of winter and spring, while long-range transport was found to play a more important role when heating activities were reduced in warmer seasons of summer. Domestic solid fuel burning can be a great health concern as studies have shown that solid fuel burning OA can be more toxic than other sources [9,15], although the toxicities of peat burning, the dominant solid fuel burning OA factor found in this region, remain poorly investigated in comparison with other solid fuels of wood and coal combustion. Considering that solid fuel burning emissions are directly linked to local heating activities, in theory, it can be reduced through the replacement of dirty fuels by cleaner fuels (e.g., gas or electricity) or using a more efficient wood stove instead of an open stove with no emission control [46]. However, in practice, emission control of solid fuel burning is challenging given that solid fuel is one of the major and cheap heating sources available for some households [35]. Even consumed by a few percentages of the households, overall air quality can deteriorate as shown in our previous case study in Dublin [47]. 

Compared to local factors, the long-range transport of secondary aerosol may require joint efforts on a regional scale, especially when secondary aerosols also have anthropogenic origins. In this study, we showed that MO-OOA and sulfate were more elevated with the easterly wind which passed over inland Ireland, although they might have origins from other European countries, e.g., the UK and France [28]. In the meantime, nitrate also shows elevated concentrations in the same wind direction. Given that nitrate can only be formed from the oxidation of NOx, a combustion byproduct, a part of MO-OOA and sulfate are likely originated from anthropogenic precursor gases or at least enhanced by anthropogenic emissions [48], even when biogenic emissions are the main precursor gases of MO-OOA and sulfate [49]. Daellenbach, et al. [9] shows SOA from anthropogenic origins is associated with higher oxidative potential than biogenic SOA. Future works will focus on investigating the origins of different SOA and get a better understanding of the corresponding formation mechanisms.

## 5. Conclusions

In this study, we performed a near real-time measurement of the NR-PM_1_ species for 6 months using an ACSM, providing insights into the seasonal variation of the chemical composition of NR-PM_1_. Source apportionment of the organic fraction was performed using the newly developed rolling PMF strategy and was compared with the conventional seasonal PMF. Moreover, the geographic origins of the primary and secondary aerosol components were investigated based on the dependence on wind speed and wind direction. The results show the bulk NR-PM_1_ concentration over the entire period was relatively low due to the frequent and relatively clean air masses, with an average of 3.0 μg m^−3^. However, episodic pollution events with concentrations up to 60.2 μg m^−3^ were also seen due to the residential heating emissions, and in particular peat burning, coupled with low wind speeds and low temperatures in winter. Peat burning OA, on average, accounted for 23% of the total OA mass in winter and contributed to up to 49% of the OA during episodic events. Additionally, HOA, wood, and coal were also associated with domestic heating, with elevated concentrations in the evening hours in winter. Even in summer, we showed that sporadic heating events also occurred with similar diurnal patterns to that in winter and spring. Two OOA subtypes were resolved, including MO-OOA and LO-OOA which were found to be the dominant OA factor during summer. On average, MO-OOA accounted for 62% of OA and was associated with long-range transport, showing a high concentration even at high easterly wind (6 m s^−1^). We also showed that MO-OOA could be from the aging of local solid fuel burning, on average, accounting for 21% of OA in winter. Similarly, LO-OOA had both local and regional origins. POA factors resolved by the two PMF strategies were very similar. However, compared to rolling PMF, the conventional seasonal PMF over-estimated LO-OOA by nearly 100% while it underestimated MO-OOA by 30%. We showed that the rolling PMF captured the temporal variation of the source profiles, presenting a more environmentally reasonable solution than conventional PMF for the secondary OA. 

## Data Availability

All data needed to evaluate the conclusions in the paper are present in the paper and/or the Supplementary Materials. Also, all data used in the study are available from the corresponding authors upon request.

## Supplementary Materials

The following supporting information can be downloaded at: https://www.mdpi.com/article/10.3390/toxics10030121/s1, Figure S1. Sampling site; Figure S2: Free PMF solution; Figure S3: time series of *f60*; Figure S4: Diurnal patterns of the meteorological parameters; Figure S5: diurnal pattern for the OA factors during weekdays and weekends; Figure S6: diurnal pattern of BC during weekdays and weekends; Figure S7: Mass spectral profile comparison; Figure S8: Distribution of the scaled residuals; Figure S9: time series of *f44*/*f43*; Figure S10: wind rose; Figure S11: Polar plot of BC; Figure S12: Polar plot of OOA factors. Table S1: Criterion list for both seasonal and rolling PMF; Table S2: Comparison of the correlation r^2^ for the MO-OOA and LO-OOA.
