# Supplementary material for: Seasonality of Aerosol Sources Calls for Distinct Air Quality Mitigation Strategies
Source: Toxics. 2022 Mar 3;10(3):121. doi: 10.3390/toxics10030121 (PMC8953366; doi:10.3390/toxics10030121)
Supplement: Supplementary file 1 [file toxics-10-00121-s001.zip › toxics-1617486-supplementary.pdf]

toxics MDPI

Supplementary Materials: Seasonality of Aerosol Sources Calls for Distinct Air Quality Mitigation Strategies

Chunshui Lin, Darius Ceburnis, Colin O'Dowd and Jurgita Ovadnevaite

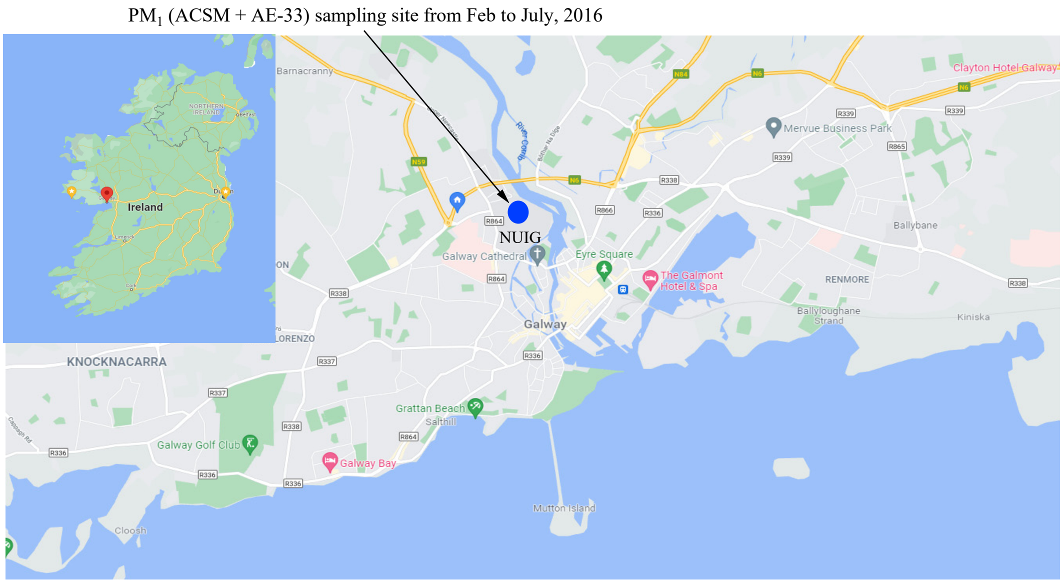

Figure S1. Sampling site (© Google Maps) at NUIG in Galway city, on the west coast of Ireland. The map is adapted from Google Maps.

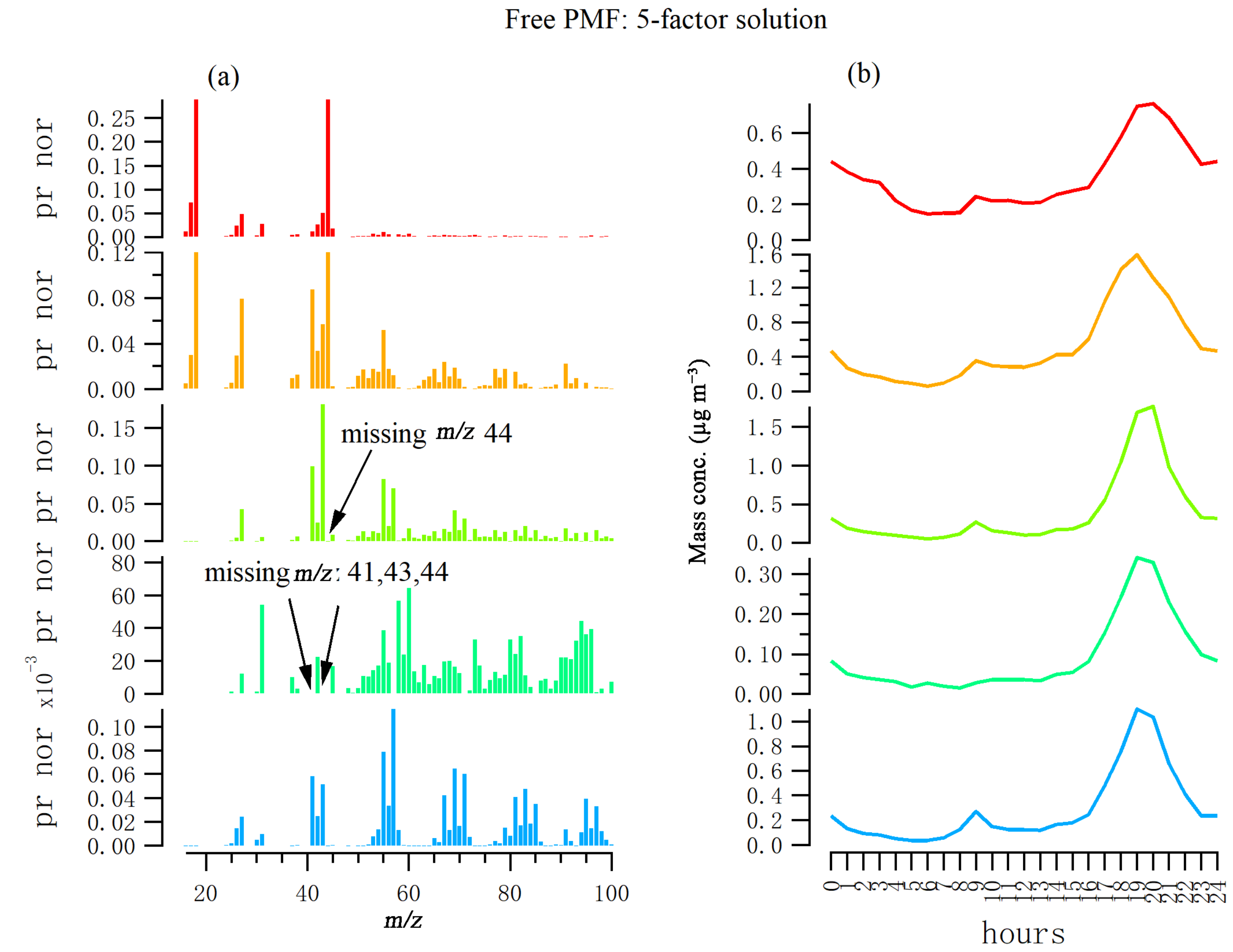

Figure S2. Mass spectral profiles for the unconstrained PMF factors (**a**) and the corresponding time diurnal patterns (**b**) in winter.

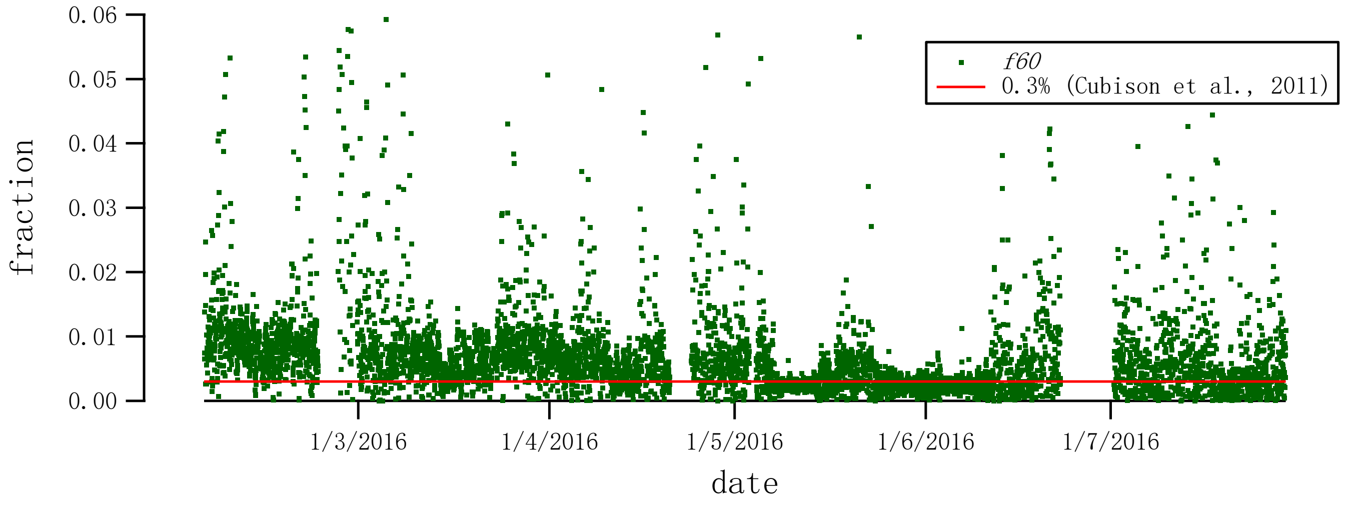

Figure S3. Time series of the measured fraction of *m*/*z* 60 (i.e., *f60*) in the total organics. The red line is the reference *f60* background from Cubison, *et al.* [1].

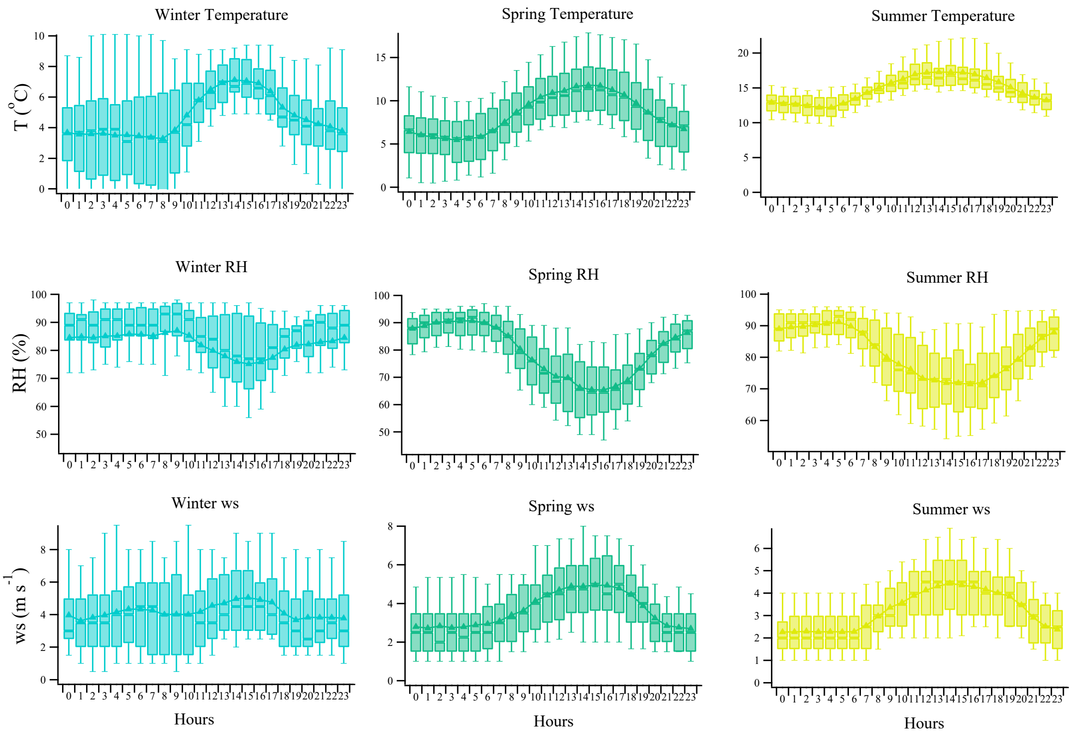

Figure S4. Diurnal patterns of the meteorological parameters in different seasons. For the diurnal plot, the triangle is the mean value; the line is the median; box is the 25th and 75th percentile; and the whisker is the 5th and 95th percentile.

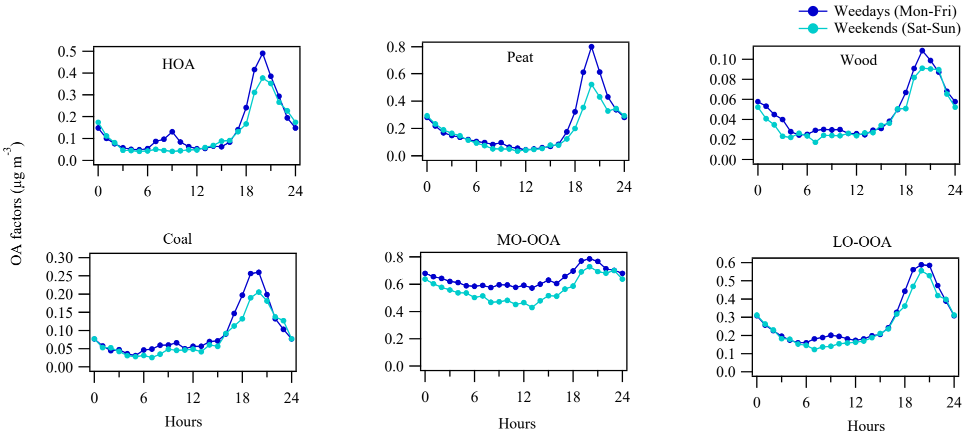

Figure S5. Averaged diurnal pattern for the OA factors during weekdays and weekends over the entire period.

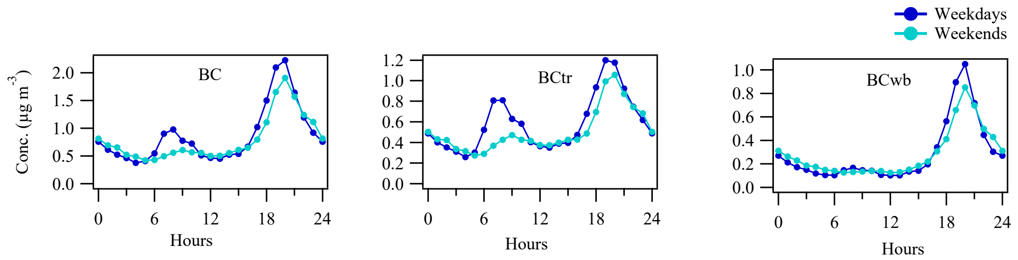

Figure S6. Averaged diurnal pattern for BC, and traffic-related BC (BCtr) and wood burning-related BC (BCwb). BCtr and BCwb were apportioned following Sandradewi, *et al.* [2] with Ångström exponent of 1 for traffic and 2 for biomass burning.

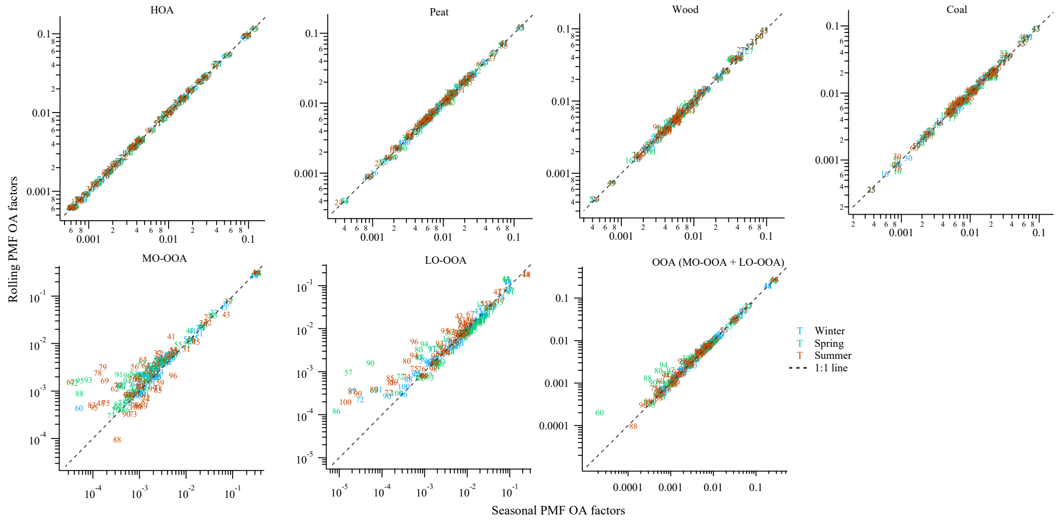

Figure S7. Mass spectral profile comparison between the rolling and seasonal PMF (log scale).

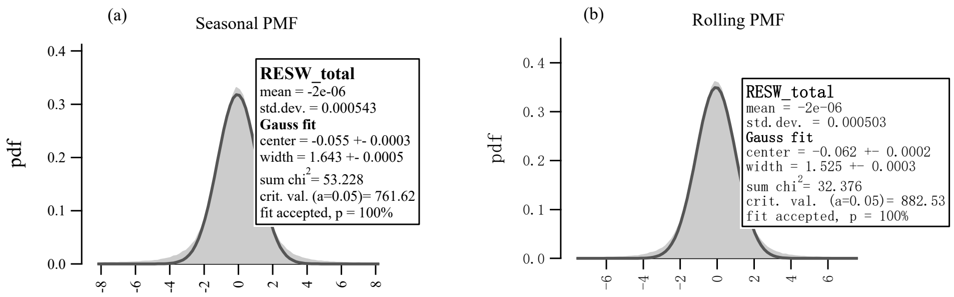

Figure S8. Distribution of the scaled residuals over the sampling period for the seasonal PMF (**a**) and rolling PMF (**b**).

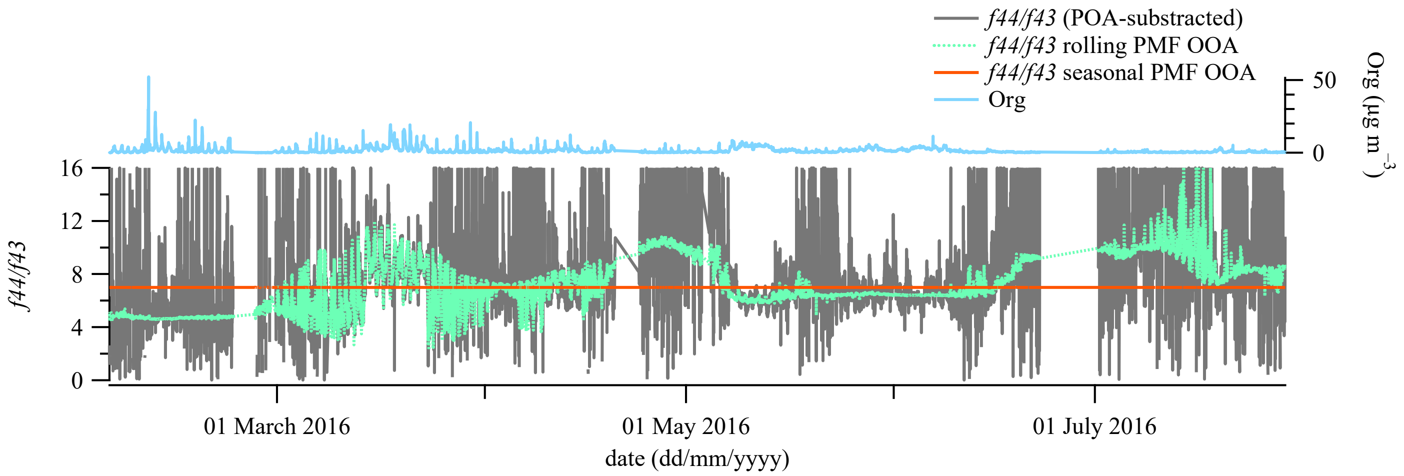

Figure S9. Time series of the measured *f44*/*f43* ratios (POA-subtracted) and OOA *f44*/*f43* for the rolling and seasonal PMF. Also shown is the time series of organic aerosol (Org). The large spikes of the *f*/*44*/*f43* were during the periods with low Org concentration (close to detection limit).

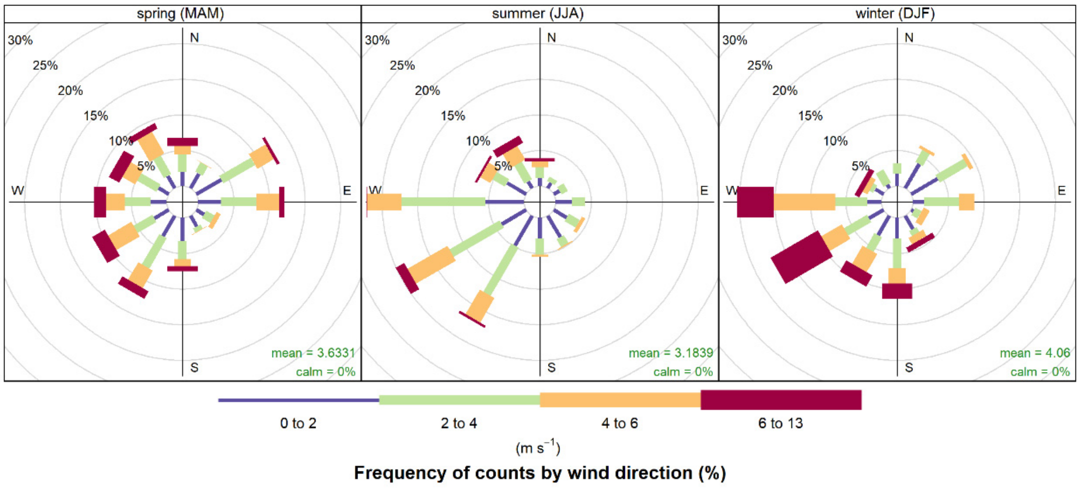

Figure S10. Frequency of counts by wind direction in different seasons. The wind rose plots were plotted using openair package with R software (version 4.1.2) [3].

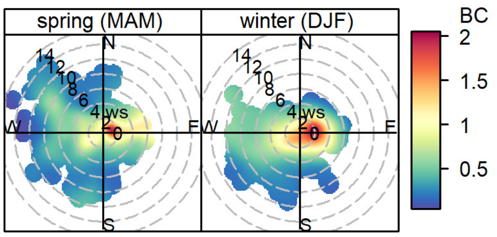

Figure S11. Polar plot of BC in spring and winter. The polar plots were plotted using openair package with R software (version 4.1.2) [3].

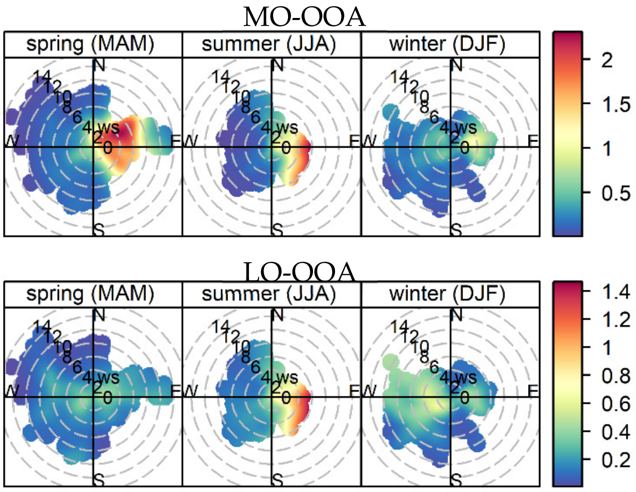

Figure S12. Polar plot of MO-OOA and LO-OOA for the seasonal PMF solution. The polar plots were plotted using openair package with R software (version 4.1.2) [3].

Table S1. Criterion list for both seasonal and rolling PMF.

Criterion	Type	Threshold
HOA vs BC*	R^2, time series during the morning rush hours (6:00-10:00)	p value ≤ 0.05
HOA + peat + wood + coal vs BC[1]	R^2, time series during the evening hours (18:00-23:00)	p value ≤ 0.05
Explained variation *m*/*z*60, wood + peat	Average, normal time series	p value ≤ 0.05
Factor_5 [44][2]	Profiles, fraction, sorting criterion	>0
Factor_5 [43]	Profiles, fraction	>0
Factor_6 [44]	Profiles, fraction	>0
Factor_6 [43]	Profiles, fraction	>0

*[1]BC measurement was only available from 05 February to 09 April 2016. During periods when BC was not available, only the rest of criterion were considered. [2] The criterion with gray background was applied as the sorting criterion for the unconstrained PMF factors

Table S2. Comparison of the correlation r^2 between the time series of MO-OOA and LO-OOA apportioned by the rolling PMF and seasonal PMF, as well as external measurements during the summer.

Correlation r^2	Rolling PMF	Seasonal PMF
MO-OOA vs. LO-OOA	0.54	0.76
MO-OOA vs. SO4	0.62	0.62
LO-OOA vs. NO3[1]	0.27	0.40
OOA vs. NH4	0.57	0.57

[1]Note that the correlation of LO-OOA vs. NO_3 is better for seasonal PMF than rolling PMF (both below 0.5). However, due to the multiple sources of the ACSM-measured NO_3 (e.g., ammonium nitrate and organic nitrates), the criteria of LO-OOA vs. NO_3 cannot be directly applied to evaluate the performance of two PMF.

References:

1. Cubison, M.J.; Ortega, A.M.; Hayes, P.L.; Farmer, D.K.; Day, D.; Lechner, M.J.; Brune, W.H.; Apel, E.; Diskin, G.S.; Fisher, J.A.; et al. Effects of aging on organic aerosol from open biomass burning smoke in aircraft and laboratory studies. *Atmos. Chem. Phys.* **2011**, *11*, 12049-12064, doi:10.5194/acp-11-12049-2011.
2. Sandradewi, J.; Prévôt, A.S.H.; Szidat, S.; Perron, N.; Alfarra, M.R.; Lanz, V.A.; Weingartner, E.; Baltensperger, U. Using aerosol light absorption measurements for the quantitative determination of wood burning and traffic emission contributions to particulate matter. *Environ. Sci. Technol.* **2008**, *42*, 3316-3323.
3. Carslaw, D.C.; Ropkins, K. openair - An R package for air quality data analysis. *Environ. Model. Soft.* **2012**, *27–28*, 52-61.
